# O-to-H Flap Reconstruction of a Central Forehead Defect Following Basosquamous Carcinoma Excision: A Case Report and Literature Review

**DOI:** 10.7759/cureus.103814

**Published:** 2026-02-18

**Authors:** Sofia Krili, Vasileios Psarras, Dimitra Koumoundourou, Maria Dimitriadou

**Affiliations:** 1 Department of Surgery, General University Hospital of Patras, Patras, GRC; 2 Department of Pathology, General University Hospital of Patras, Patras, GRC

**Keywords:** basosquamous carcinoma, central forehead defect, h-plasty, local flap reconstruction, o-to-h flap

## Abstract

Basosquamous carcinoma (BSC) is a rare, aggressive non-melanoma skin cancer that exhibits histopathological features of both basal and squamous cell carcinoma. Surgical excision remains the first-line treatment; however, standardized guidelines for its management and reconstruction are lacking. Central forehead defects pose reconstructive challenges due to limited tissue mobility, high tension, and both aesthetic and functional significance.

An 88-year-old male presented with a 2.6 x 2.4 cm ulcerative lesion on the central forehead, histopathologically diagnosed as infiltrative BSC. Under local anesthesia, a wide surgical excision was performed, including a small periosteal segment. The resulting 4.6 x 4.4 cm defect was reconstructed using an O-to-H flap. Horizontal incisions were designed parallel to relaxed skin tension lines, Burrow’s triangles were excised, and vertical sutures converted the O-shaped defect into an H configuration. Meticulous hemostasis was achieved, and closure was completed with 5-0 nylon sutures. At the four-month follow-up, the patient demonstrated excellent functional and aesthetic outcomes. The scar healed well, sensory function was preserved, and there was no evidence of local recurrence.

Local flap reconstruction, particularly H-plasty, provides reliable coverage for central forehead defects, with favorable aesthetic results and low donor-site morbidity. Careful dissection minimizes the risk of supratrochlear and supraorbital nerve injury.

The O-to-H flap represents a simple and effective option for central forehead reconstruction following BSC excision, achieving optimal functional and cosmetic outcomes. Further studies are needed to establish standardized management and reconstructive strategies for BSC.

## Introduction

The forehead is a non-hair-bearing and aesthetically significant region that contains the frontalis muscle and the frontal branch of the facial nerve, as well as the supratrochlear and supraorbital nerves and their accompanying vessels. Defects in this area may result from various etiologies, including trauma, electric burn, tumor resection, radiation, and infection [1]. Furthermore, the scalp constitutes a common site for skin cancer owing to its constant and direct exposure to sunlight [2].

Reconstruction of central forehead defects is challenging due to the limited availability of local extensible tissue and the high aesthetic importance of the region [3]. The central forehead plays a critical role in facial expression and symmetry; therefore, reconstruction should preserve brow symmetry and the natural hairline. Optimal cosmetic outcomes are achieved by orienting closure lines along the relaxed skin tension lines or the hairline to minimize scar visibility [3]. Various local flap techniques have been described to address these challenges, each presenting limitations in mobility, tension distribution, and aesthetic harmony [3].

Basosquamous carcinoma (BSC) is a rare yet aggressive nonmelanoma skin cancer exhibiting characteristics ranging from basal cell carcinoma (BCC) to squamous cell carcinoma (SCC) [4]. It typically presents as a persistent nodular lesion that may gradually evolve into an ulcer, representing its most characteristic clinical manifestation. BSC usually arises in sun-exposed areas of the head and neck, and its optimal management remains a subject of ongoing debate [4].

We report the case of an 88-year-old male with an infiltrative basosquamous carcinoma of the forehead, who underwent wide surgical excision. The resulting defect was reconstructed using an O-to-H-flap. At the four-month follow-up, the patient demonstrated excellent functional and aesthetic outcomes.

## Case presentation

**Figure 1:**
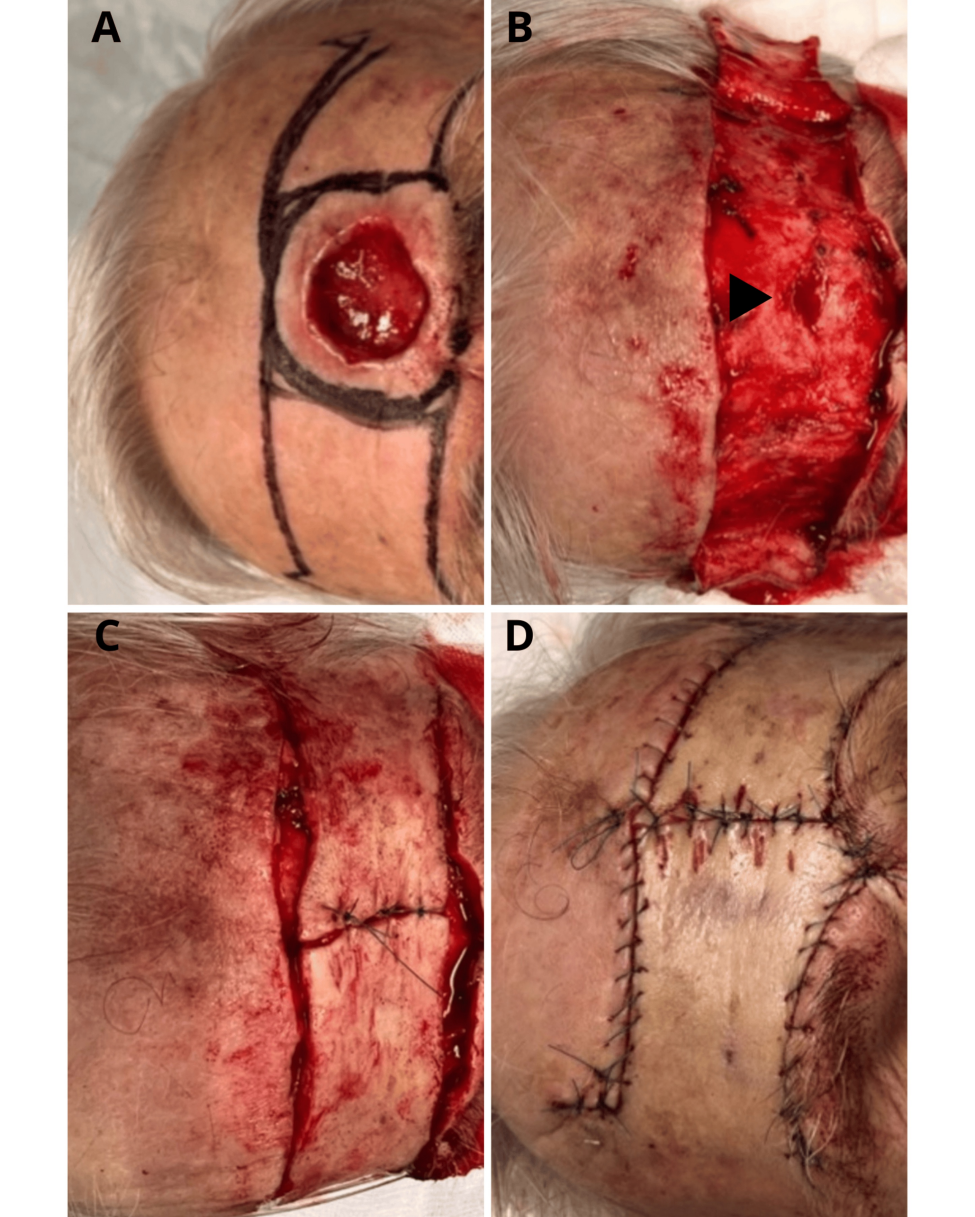
O-to-H flap reconstruction of the central forehead lesion. A) Preoperative image of the central forehead ulcerative lesion, with the planned surgical margins and the O-shaped defect delineated. B) Intraoperative image following lesion excision and mobilization of the opposing rectangular flaps; focal periosteal infiltration is indicated by a black arrowhead. C) Intraoperative image after placement of vertical sutures between the two flaps, illustrating the resulting H-shape. D) Final outcome after completion of the O-to-H flap reconstruction.

**Figure 2:**
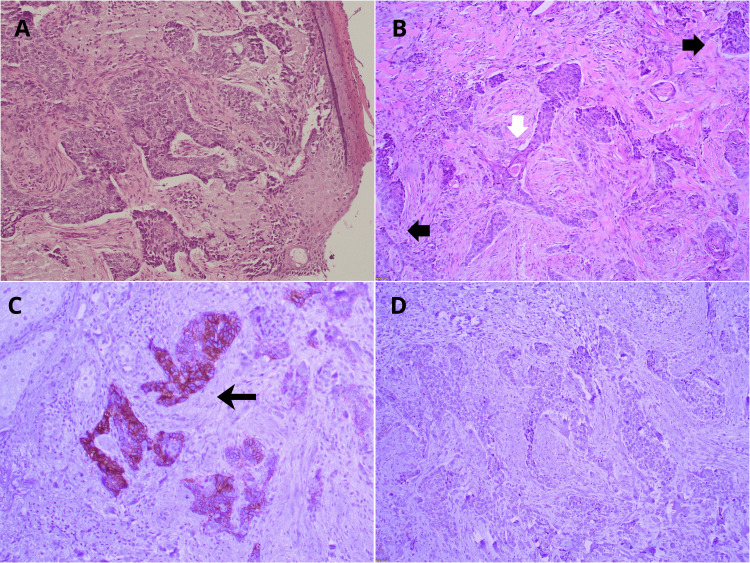
Representative histopathological and immunohistochemical images of the central forehead lesion. A) Hematoxylin and eosin (H&E)-stained section (x100) from the incisional biopsy demonstrating nests of basaloid cells consistent with basal cell carcinoma differentiation. B) H&E-stained section (x100) from the incisional biopsy showing mixed tumor differentiation: the central white arrow indicates areas of squamous differentiation, while the black arrows highlight the basaloid tumor cell nests, confirming the coexistence of basal and squamous components consistent with basosquamous carcinoma. C) Immunohistochemical staining for BerEP4 (x100) performed on the excisional biopsy specimen demonstrating strong positive staining (black arrow) in the basaloid tumor component. D) Immunohistochemical on the excisional biopsy specimen showing absence of immunoreactivity, corresponding to areas of squamous cell differentiation.

An 88-year-old male patient presented to the Plastic and Reconstructive Surgery outpatient clinic with a progressively enlarging nodule that evolved into a non-healing ulcer over the central forehead, persisting for the last two years. The ulcerative lesion measured 2.6 x 2.4 cm and was circular in shape (Figure 1A). The patient’s medical history included cardiac comorbidities with recent pacemaker implantation and type II diabetes mellitus, precluding the use of general anesthesia. The diagnosis of infiltrative basosquamous carcinoma was made through incisional biopsy (Figure 2). The patient declined preoperative computed tomography (CT) imaging, preventing precise assessment of the lesion’s extent and potential lymph node involvement. 

Under local anesthesia, a wide local excision of the lesion was performed with a 10 mm margin circumferentially. The base of the excision extended to the periosteum, from which a small segment was also removed as it appeared to be infiltrated intraoperatively. The final defect size measured 4.6 x 4.4 cm. The central forehead defect was reconstructed using an O-to-H flap, a local double-advancement flap technique. The initial circular (O-shaped) defect was converted into two opposing rectangular advancement flaps by creating an upper and a lower horizontal incision, planned parallel to and concealed within the natural transverse forehead relaxed skin-tension lines (RSTLs) formed by the frontalis muscle (Figure 1B). Each incision measured approximately twice the lesion’s diameter, with its midpoint aligned to the center of the defect. The Burrow’s triangles in the lateral upper forehead were then excised to remove redundant tissue. The opposing flaps were elevated in the subcutaneous plane and advanced towards the defect. Then, vertical sutures were placed between the two flaps, completing the conversion of the O-shaped defect into an H configuration (Figures 1 C, 1D). Meticulous hemostasis was achieved using bipolar cautery, and skin closure was performed using continuous and interrupted 5-0 Nylon sutures to minimize scarring and maintain contour symmetry.

Histopathological examination of the lesion revealed an infiltrative basosquamous carcinoma involving the subcutaneous tissue, underlying muscle fibers, and focal areas of the periosteum segment. No perineural or lymphovascular invasion was identified. Immunohistochemical analysis further supported the diagnosis and delineated the distinct basaloid and squamous tumor components, confirming the lesion as basosquamous carcinoma (Figure 2). Following multidisciplinary consultation, postoperative radiotherapy was recommended; however, the patient declined adjuvant treatment. 

**Figure 3:**
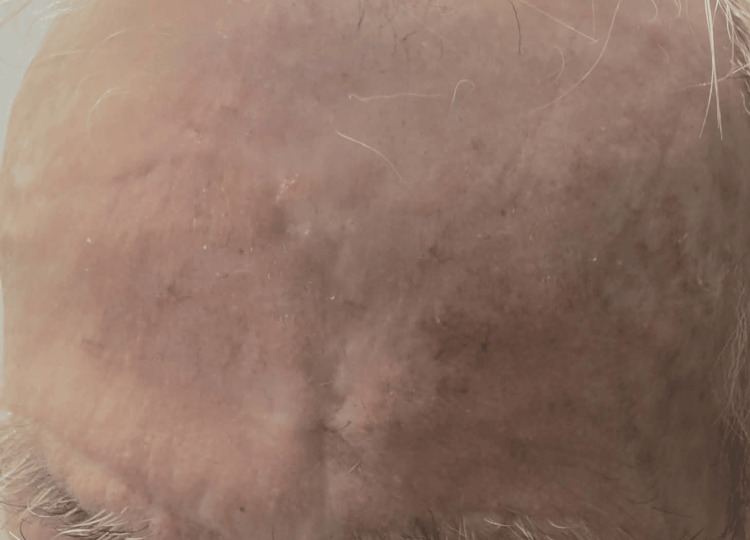
: Postoperative image of the patient (3/4 view) at four-month follow-up.

At the four-month follow-up, the patient remained asymptomatic with excellent aesthetic and functional outcomes. The scar healed favorably along the RSTLs; there were no sensory deficits, the hairline and eyebrow position were well preserved, and there was no evidence of local recurrence or postoperative complications (Figure 3).

## Discussion

Basosquamous carcinoma (BSC), also referred to as metatypical carcinoma (MTC), is a rare non-melanoma skin cancer that exhibits both squamous and basal cell differentiation [5]. This tumor should be considered as a distinct clinopathologic entity, with its own biological behavior and histopathological characteristics. Representing an intermediate form of basal cell carcinoma (BCC) and squamous cell carcinoma (SCC), MTC clinically and morphologically resembles BCC but demonstrates more aggressive behavior and higher potential for local recurrence and metastasis [5]. Accurate distinction of MTC from the group of basaliomas is therefore essential, as its clinical and histologic similarity to BCC can complicate diagnosis [5]. The reported incidence of BSC ranges from 1.7 to 2.7% [6].

As with BCC and SCC, surgical excision constitutes the first-line treatment for BSC [7], while several studies have highlighted the efficacy of Moh’s micrographic surgery and adjuvant radiotherapy in achieving lower recurrence rates [8,9]. Standardized therapeutic guidelines for the management of BSCs have not yet been established; however, Jordan W. Oldbury et al. proposed a unit-based protocol derived from both literature review and their institutional experience to guide the treatment and follow-up of patients with BSC (Table 1) [10].

**Table 1:** Proposed unit protocol for basosquamous carcinoma (BSC). A proposed unit protocol designed to guide treatment and follow-up for patients diagnosed with BSC. From Oldbury JW, Wain RAJ, Abas S, Dobson CM, Iyer SS. J Skin Cancer. 2018; 2018:6061395 [10]. Licensed under CC BY 4.0.

Proposed unit protocol for BSC
At the time of diagnostic surgery:
(i) Punch or incision biopsy of suspicious lesion or,
(ii) If easily excisable measure and mark peripheral margins as per BCC guidance, i.e., 4mm.
(iii) Always ensure next clear anatomical plane is reached at the deep margin.
(iv) If the lesion is suspicious of SCC, excise according to appropriate (EDF/BAD) SCC guidelines.
(v) In the case of confirmed BSC preoperatively, MMS should be offered where available.
At the first clinic review:
(i) Explain difference between BSC and BCC i.e. whilst BSC has the potential for regional and distant metastasis, this is uncommon in pT1 and pT2 lesions, without deep invasion of the tumor.
(ii) Full examination of excision site and regional nodes.
Subsequent follow-up:
(i) All patients to have full examination of excision site and regional nodes.
(ii) Completely excised pT1 and pT2 BSC - follow-up 3-4 monthly for 24 months, then discharge if well.
(iii) Incompletely excised pT1 and pT2 BSC - offer wider excision or radiotherapy after skin MDT discussion, follow-up 3-4 monthly for 24 months, then discharge if well.
(iv) All pT3 and pT4 BSC, and those with invasion into deep structures e.g. fascia, muscle, cartilage or bone, require skin MDT discussion, appropriate treatment and follow-up 3-4 monthly for 3 years, and then 6 monthly to total of 5 years follow-up.

There is no clear consensus regarding the optimal excision margins for BSC. In their study, Goldberg and Griffith recommended surgical margins of 2 to 5 mm for BCCs and up to 10 mm for infiltrative lesions [11,12]. In addition, Murgia et al. recommended wider surgical margins for BSCs compared to those used for low-risk BCCs, given their infiltrative growth pattern [7].

Reconstruction of defects in sun-exposed areas, such as the face, presents particular challenges due to their prominent location and important aesthetic and functional role [13]. Specifically, the forehead, which constitutes approximately one-third of the face, is especially demanding for reconstruction because of its relatively high tissue tension and limited excess skin available for mobilization [14]. Moreover, the forehead includes several critical anatomical structures, including motor and sensory nerves, making preservation of function a key surgical objective. Essential considerations in forehead reconstruction include maintaining eyebrow position and concealing incision lines within the relaxed skin tension lines (RSTLs), hair-bearing areas, or between cosmetic subunit junctions [15]. Primary closure represents the simplest reconstructive option; however, when it is not feasible, alternative techniques, such as local tissue flaps, skin grafting, and second-intention healing, should be considered [14].

Local flaps closely match the architecture of the recipient site and are associated with low donor-site morbidity, yielding superior aesthetic outcomes. According to Besley et al., scalp defects up to 50 cm² can be managed with local flaps and primary closure of the donor site, whereas defects ranging from 50 to 200 cm² typically require local scalp flaps with skin grafting at the donor area [4]. Free tissue transfer is generally indicated for very large defects, those lacking periosteum, or when it is an associated calvarial defect [4].

A variety of local flap techniques have been reported for reconstructing central forehead defects, such as subcutaneous V-Y pedicled flaps [16,17], A-to-T rotation-advancement flaps for defects situated near the hairline [18], and multiple hatchet flaps [19]. Double opposing rectangular advancement flaps, or H-plasty, represent an old-established technique in the plastic surgeon’s repertoire, but it is not frequently emphasized in the context of forehead reconstruction [18]. In the series reported by Rose et al., the H-plasty technique has become a preferred method for such defects, primarily for cosmetic reasons [20]. The transverse scars are easily concealed within the forehead rhytids, and the vertical scar length is minimized by matching it to the vertical dimension of the defect [20]. Rose et al. recommend using this technique for defects up to 30 mm in diameter, raising flaps with a length-to-breadth ratio up to 2:1 without compromising the distal portions [20]. Alternative local flaps mentioned above often result in more significant vertical scars, oblique scars that are less well hidden within skin creases, or disruption of the hairline [20]. Transverse incisions may endanger the supratrochlear and supraorbital nerves, especially when flap elevation extends into the subgaleal plane; however, careful and precise dissection can significantly minimize this risk [19]. Although local cutaneous flaps are often accompanied by some degree of sensory alteration, several studies indicate that such symptoms are generally well tolerated by patients [20].

Taking all the considerations into account, we employed H-plasty for the reconstruction of a central forehead defect with a maximum diameter of 46 mm, resulting from a wide surgical excision of a basosquamous carcinoma. The aesthetic and functional outcomes were highly satisfying, as the scar healed well at the four-month follow-up and there were no sensory deficits or evidence of tumor recurrence in the area.

## Conclusions

Given the lack of standardized treatment protocols for BSC, prospective comparative studies are required to delineate the most effective management strategies. Surgical intervention, particularly wide excision and Mohs micrographic surgery, remains the modality of choice for many practitioners. Reconstruction of facial defects varies depending on tumor size and location, often necessitating different operative techniques. This paper highlights the efficacy of H-plasty for central forehead reconstruction, including defects greater than 30 mm, underscoring it as a simple and reliable technique that provides excellent functional and aesthetic outcomes.
